# Management of psychiatric disorders in the armed forces: exemptions and changing specialty

**DOI:** 10.1192/j.eurpsy.2025.1833

**Published:** 2025-08-26

**Authors:** N. Khaterchi, S. Kamoun, H. Ziedi, A. Amri

**Affiliations:** 1 Occupational medicine, Military occupational health center, Tunis, Tunisia

## Abstract

**Introduction:**

Military personnel, faced with stressful and often extreme situations, can develop psychiatric disorders which require specific management to maintain both their well-being and the operational effectiveness of units. These disorders pose significant challenges for the assessment of their fitness for military service.

**Objectives:**

To study the impact of psychiatric pathologies on fitness for military service.

**Methods:**

This was a retrospective study of a descriptive nature, from January 1, 2023 to August 30, 2024, which was conducted among military personnel referred to the occupational pathology consultation at the Military Center for Occupational Medicine and Professional Safety in Tunis to assess their fitness for duty due to psychiatric disorders.

**Results:**

During the study period, 42 patients were included, with a mean age of 38 ± 10 years, all male. The study population was divided into non-commissioned officers (45.2%), unlisted men (40.5%) and officers (14.3%), 44.5% of whom belonged to the army, 19.4% to the navy, 11.1% to the air force and 25% to the joint services. The ranks most affected by psychiatric pathologies were the first-class soldier at 21.4%, the master corporal and the warrant officer at 16.7% each. The most common specialties were infantry and transport at 28.6% and 11.9% respectively. The most common work positions were administrative officer (19.1%) and infantryman (9.5%). The predominant pathologies were anxiety-depressive syndrome in 31% of cases, depression in 26.2%, anxiety disorder in 14.3% and post-traumatic stress disorder in 11.9%. Eighty-one percent were exempted from carrying weapons, 57.1% from guard duty and 2.4% from field trips, with an average exemption duration of 5 months. All requests for exemption were accepted, of which 22% led to a reform file, 14% to psychiatric care, and 5% to a notice of fitness for military service. A change of specialty was indicated in six cases, all of them to an administrative position.

**Conclusions:**

Psychiatric disorders strongly affect fitness for military service, leading to exemptions and changes of specialty. Enhanced psychological care and rigorous follow-up are essential to preserve the operational effectiveness and well-being of military personnel.

**Disclosure of Interest:**

None Declared

